# Functional Changes in the Human Auditory Cortex in Ageing

**DOI:** 10.1371/journal.pone.0116692

**Published:** 2015-03-03

**Authors:** Oliver Profant, Jaroslav Tintěra, Zuzana Balogová, Ibrahim Ibrahim, Milan Jilek, Josef Syka

**Affiliations:** 1 Department of Auditory Neuroscience, Institute of Experimental Medicine, Academy of Sciences of the Czech Republic, Prague, Czech Republic; 2 MR Unit, Department of Diagnostic and Interventional Radiology, Institute for Clinical and Experimental Medicine, Prague, Czech Republic; 3 Department of Otorhinolaryngology and Head and Neck Surgery, 1^st^ Faculty of Medicine, Charles University in Prague, University Hospital Motol, Prague, Czech Republic; Kyoto University, JAPAN

## Abstract

Hearing loss, presbycusis, is one of the most common sensory declines in the ageing population. Presbycusis is characterised by a deterioration in the processing of temporal sound features as well as a decline in speech perception, thus indicating a possible central component. With the aim to explore the central component of presbycusis, we studied the function of the auditory cortex by functional MRI in two groups of elderly subjects (>65 years) and compared the results with young subjects (<lt;30 years). The elderly group with expressed presbycusis (EP) differed from the elderly group with mild presbycusis (MP) in hearing thresholds measured by pure tone audiometry, presence and amplitudes of transient otoacoustic emissions (TEOAE) and distortion-product oto-acoustic emissions (DPOAE), as well as in speech-understanding under noisy conditions. Acoustically evoked activity (pink noise centered around 350 Hz, 700 Hz, 1.5 kHz, 3 kHz, 8 kHz), recorded by BOLD fMRI from an area centered on Heschl’s gyrus, was used to determine age-related changes at the level of the auditory cortex. The fMRI showed only minimal activation in response to the 8 kHz stimulation, despite the fact that all subjects heard the stimulus. Both elderly groups showed greater activation in response to acoustical stimuli in the temporal lobes in comparison with young subjects. In addition, activation in the right temporal lobe was more expressed than in the left temporal lobe in both elderly groups, whereas in the young control subjects (YC) leftward lateralization was present. No statistically significant differences in activation of the auditory cortex were found between the MP and EP groups. The greater extent of cortical activation in elderly subjects in comparison with young subjects, with an asymmetry towards the right side, may serve as a compensatory mechanism for the impaired processing of auditory information appearing as a consequence of ageing.

## Introduction

Ageing affects the central nervous system in several ways, resulting in the atrophy of grey and white matter [1], changes in the levels of cortical metabolites and in functional deterioration leading to cognitive decline. The latter is even more pronounced in the case of Alzheimer’s disease, mild cognitive impairment or dementia, which are more prevalent in the ageing population [2]. Elderly individuals with the above-mentioned pathologies are also more susceptible to develop age related hearing loss, presbycusis [3, 4].

In general, hearing loss is one of the most prevalent sensory deficits that affect the elderly population. Currently, over 30–35% of adults between 65–75 years of age and 40–50% of adults above 75 years of age [5–8] suffer from this condition. In addition to increasing life expectancies, presbycusis is becoming an increasingly relevant social healthcare and economic problem [9].

The dominant pathological changes in presbycusis are observed in the auditory periphery, specifically in the cochlea[10, 11].The impairment of the inner ear results from life-long exposure to noise insults, ototoxic agents, otologic and systemic diseases, and mostly from a combination of all these factors accompanied by genetic susceptibility to the condition [12].

Peripheral pathologies affect hearing predominantly at frequencies above 1 kHz, which are important frequencies for speech processing. Such deterioration can be compensated by auditory hearing aids, but whilst this approach can improve the hearing threshold to near the physiologic level [13], there is a clear deficit in speech understanding; this is particularly pronounced under altered acoustic conditions, such as the presence of background noise [14]. One of the possible explanations for decreased speech understanding in the elderly is the inability to clearly detect temporal cues [15]. The processing of temporal cues takes place mainly in the auditory cortex (AC) [16], which indicates a possible central component of presbycusis. It is also dependent on the audibility of high frequency sounds, therefore temporal processing is decreased in listeners with high frequency hearing loss [17, 18]. The dominant question in understanding the central component of presbycusis is whether it is a consequence of peripheral hearing loss, central changes independent of the auditory periphery, a combination of both, or a result of other cognitive deficits.

The aim of our experiments was to explore the difference in activations of the auditory cortex by simple acoustical stimuli in old and young subjects and observe how the use of simple acoustic stimulation reduced the possible effect of age related cognitive decline on the central processing of sounds. In addition, with the aim to reveal how the hypo-functional inner ear contributes to age related central changes of AC function, we employed two groups of old subjects, one with mild presbycusis and one with expressed presbycusis. On all subjects, we performed a detailed audiological examination to evaluate the function of the inner ear as well as their speech perception in noise. For assessing the function of the AC, fMRI focused on the AC was used during pink noise stimulation.

## Material and Methods

### Subjects

Forty-eight subjects were examined in this study and were divided into 3 groups based on their age and hearing thresholds (examined by pure tone average and compared with the ISO 7029 norm specified for their age): 15 elderly subjects with a mild state of presbycusis (MP; 4 men and 11 women) between the ages of 65–72 (mean age ± SEM; 67.9±0.49), 15 elderly subjects with expressed presbycusis (EP; 9 men and 6 women) between the ages of 64–79 (mean age ± SEM 70.7±1.37) and 18 young subjects (11 men and 7 women) between 22 and 30 (mean age ± SEM 23.75±0.38) were used as controls (YC). All of the examined subjects declared they had had no previous otologic surgery, vestibular lesion, tinnitus, severe head trauma, lesion of the facial nerve, disorder of the cervical spine, self-reported central nervous system disorder, or contraindication for safe MRI scanning. None of the subjects were musical professionals or played musical instruments regularly. Otoscopic examination with the removal of cerumen and confirmation of an intact tympanic membrane were preformed on all subjects. The examination procedures and the informed consent were approved by the Ethics Committee of the University Hospital Motol, in Prague and the Ethics Committee of Institute for Clinical and Experimental Medicine. All participants provided their written informed consent to participate in this study; signed written consents are stored at the Department of Auditory Neuroscience, IEM CAS.

### Assessment of the auditory function

Pure tone audiometry in the extended frequency range was used to divide volunteers into groups. Oto-acoustic emissions and speech audiometry in a noisy background were used to further characterize the 3 groups. All audiometric investigations were performed in a sound attenuated chamber, with measurements performed monaurally and the ears of each subject tested successively.

**Tympanometry.** Tympanometry was performed with an Interacoustics AZ26 Tympanometer to confirm optimal middle ear conditions and intact tympanic membrane. All volunteers had type A tympanometric curves, i.e. their middle ear function was normal.

**Pure tone audiometry.** For pure tone audiometry (PTA) over an extended frequency range from 125 Hz to 16 kHz, a Madsen Orbiter 922, Version 2, audiometer was used and calibrated by the Czech Institute of Metrology according to Czech State Norm and the European Standards EN ISO 389-1 and EN ISO 389-5. Acoustical signals were delivered monaurally via Sennheiser HAD 200 high-frequency headphones. Audiograms were measured in one-octave steps at frequencies ranging from 125 Hz to 8 kHz and then at 10, 12.5 and 16 kHz; hearing thresholds were detected with a resolution of 5 dB steps; the contralateral ear was masked if necessary. Bone conduction testing was performed to exclude possible elevated thresholds due to conductive hearing loss.

**Fig 1:**
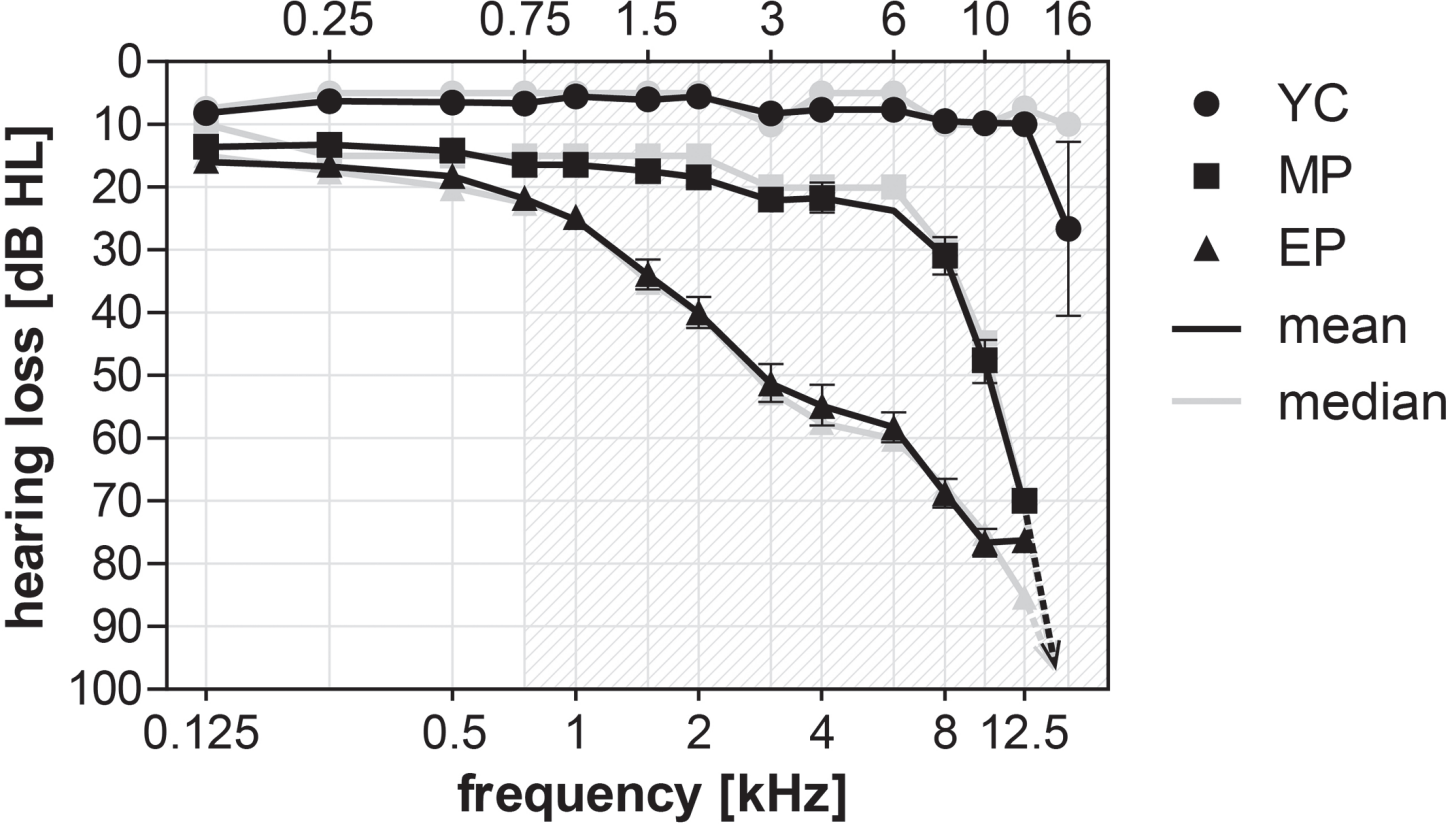
Results of the pure tone audiometry in the extended high-frequency range in all three groups. Data shown as mean±SEM and medians (in case of no hearing sensation 110 dB was set as the threshold).

The YC group displayed normal hearing with thresholds not exceeding 20 dB above the values typical for their age (Fig. 1). The MP group showed physiologic auditory thresholds (≤20 dB) comparable to the young control group at frequencies up to 4 kHz. There was a small, but steady, increase in the thresholds at higher frequencies with 70 dB hearing loss at 12 kHz, whilst hearing was preserved at 16 kHz only in 4 volunteers. The EP group’s threshold increase exceeded the physiologic range starting at 1 kHz and reached 76 dB hearing loss at 12.5 kHz with no hearing at 16 kHz. No significant difference between the left and right ears was found; therefore the thresholds of both ears were merged together.

**Oto-acoustic emissions.** Oto-acoustic emissions (OAEs) were measured with an ILO 96 Otoacoustic Analyzer system (Otodynamics, Ltd) with a B-type probe, controlled with ILO 96 software, Version 5. Standard procedures were undertaken to avoid resonance peaks in the frequency spectrum of the stimulus. For the transiently evoked OAEs (TEOAEs), the stimulus used was the conventional, broadband nonlinear click (0.5–6.0 kHz), with stimulus gain adjusted to 81 dB peak. The stimuli used for eliciting distortion product OAEs (DPOAEs) at 2f_1_-f_2_ were two primary tones (f_2_/f_1_ = 1.22 with L_1_ = 65 and L_2_ = 55 dB SPL).

**Fig 2:**
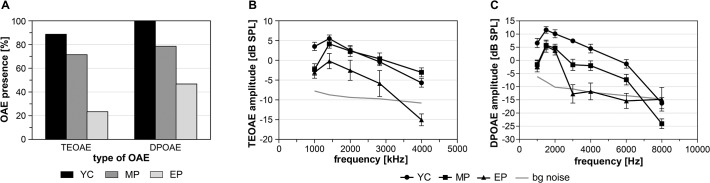
TEOAEs and DPOAEs measured in all three groups. A. Presence of OAEs in measured ears from all three groups. B. Frequency selective TEOAEs (mean amplitudes with SEM). C. Frequency selective DPOAEs (mean amplitudes with SEM).

TEOAEs were present in 89% of ears in YC with overall levels of 10.4±0.7 dB SPL (Fig. 2A). In MP, TEOAEs were present in 71.4% of ears with an overall level of 9.6±0.9 dB SPL. In EP, TEOAEs were present in 23.3% of ears with an overall level of 5±1.6 dB SPL. Frequency specific TEOAEs were present at all measured frequencies (1–4 kHz) in YC and MP, however in EP, TEOAEs were present above background noise only at low frequencies (1, 1.4 and 2 kHz) (see Fig. 2B). DPOAE were present in 100% of YC ears, 78.6% of MP ears and 46.6% of EP ears (Fig. 2A). Frequency specific DPOAEs showed average amplitudes clearly above the background noise at all measured frequencies except for 8 kHz in YC and MP ears. Amplitudes were significantly higher in YC than in MP at all measured frequencies, except for at 6 kHz (for 1 kHz (p = 0.005, F(2,69) = 7.443), 1.5 kHz (p = 0.0179, F (2,69) = 5.399), 2 kHz (p = 0.0371 F(2,69) = 4.799), 3 kHz (p = 0.0006, F(2,69) = 29.33), and 4 kHz (p = 0.0482, F(2,69) = 13.77); p<0.01 for; p<0.001 for; one way ANOVA, Tukey’s correction). DPOAEs in EP were present only at frequencies ≤2 kHz, at which the amplitudes were comparable with MP (Fig. 2C). The comparison of OAE amplitudes (TE response, DP level) between left and right ears in each group did not show any significant differences (unpaired t-test), although there was a tendency to change from right over left ear dominance in YC (mean difference in dB SPL -0.625, p = 0.68 for TE response and -1.057, p = 0.52 for DP level, negative value means right ear dominance) to left over right ear dominance in both elderly groups (in dB SPL, MP: TE response -0.156, p = 0.93, DP level 0.745, p = 0.759; MP: TE response 2.33, p = 0.497, DP level 2.026, p = 0.493).

**Speech audiometry.** Speech audiometry in quiet conditions was examined using a clinical Interacoustics AC40 audiometer with Sennheiser HDA 200 headphones. Speech audiometry in noisy conditions was examined using a clinical Madsen Orbiter 922, Version 2, audiometer with Sennheiser HDA 200 headphones. Speech discrimination scores (SDS) for sentences were determined using a standard CD recording of Czech sentence audiometry according to Dlouhá and Vokřál [19] with background babble noise levels of 65 and 70 dB.

**Fig 3:**
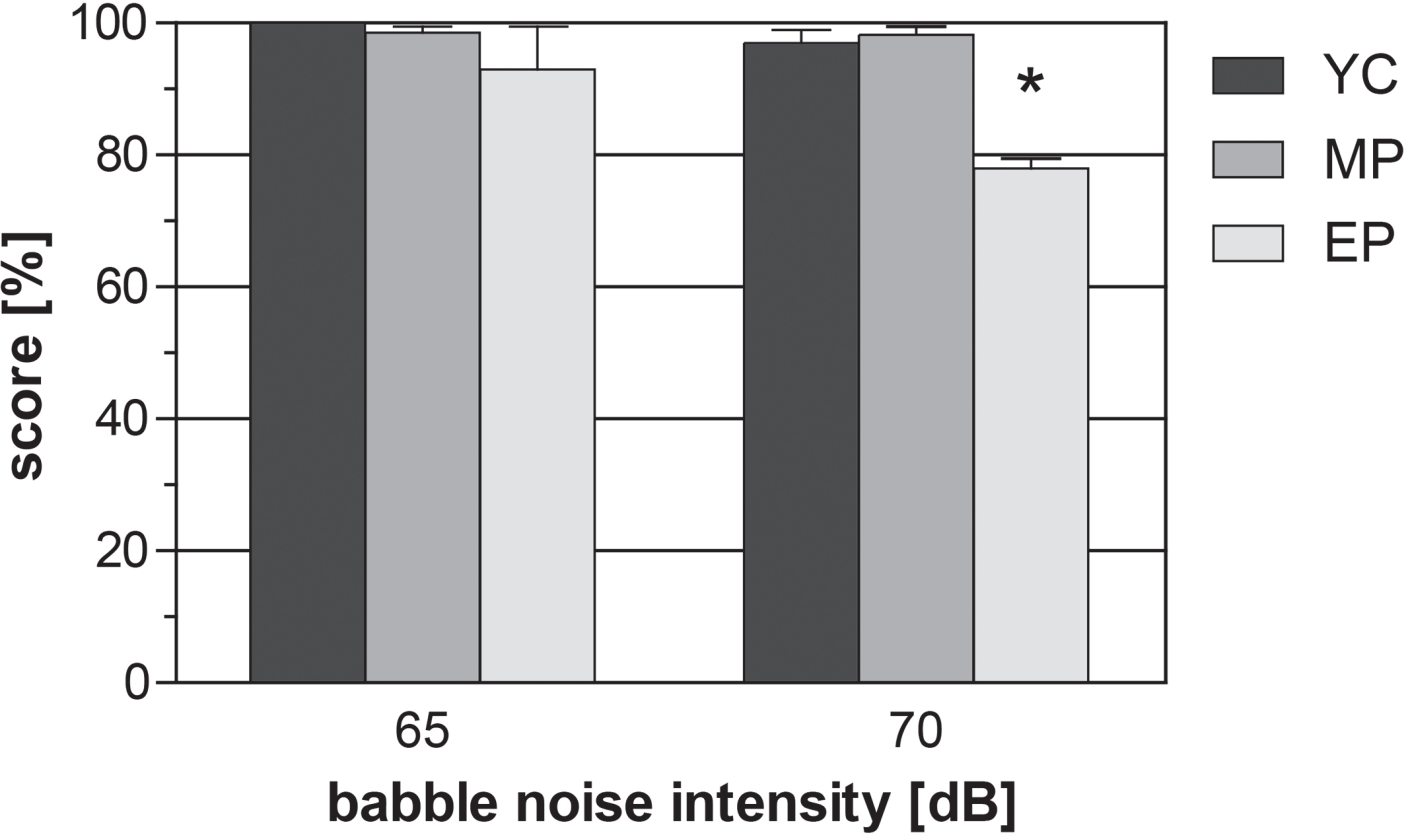
Speech discrimination score (SDS) in background babble noise. Level of babble noise was set at 65 and 70 dB. Average values with SEM, one way ANOVA, Tukey’s correction, * p<0.05.

Volunteers from all three groups scored above 90% with background noise (BN) at 65 dB (YC 100%, MP 99% and EP 92%). The SDS with a BN of 70 dB caused only a minimal decline in YC (97%) and MP (98%) groups, however there was a significant fall (p<0.001, F(5,90) = 7.908: EP vs. YC, MP; one way ANOVA, Bonferroni correction) in the SDS in the EP group (74%) (Fig. 3). This decrease was also significant when the SDS in babble noise was compared at 65 and 70 dB levels for the EP group (p = 0.0095, F(5,90) = 7.908, one way ANOVA, Bonferroni correction).

**Fig 4:**
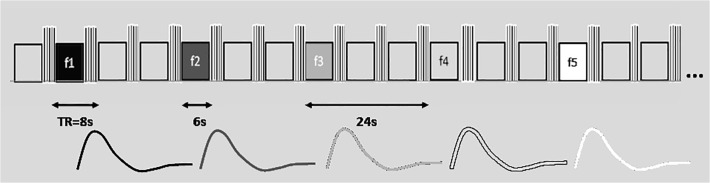
Stimulation scheme used for the recording of evoked activity for each separate frequency range during one single measurement. Each event of acoustic stimulation lasted 6 s and was followed by 3 scans (TR = 8s) without stimulation. The block shown here was repeated 10 times. Diagram shows the model used for statistical evaluation (HRF for single 6 s stimulus).

**FMRI examination of the auditory cortex.** Functional MRI examinations were performed on a Siemens Trio 3T scanner (Siemens, Erlangen, Germany) using a 12-channel head coil and a GRE-EPI sequence: TE = 30 ms, TR = 8 s, flip angle = 90°, voxel size of 3x3x3 mm^3^, 30 slices (thickness of 3 mm). An event-related design with sparse data acquisition was used. For acoustic stimulation third-octave pink noise centered at five different frequencies 350 Hz, 700 Hz, 1.5 kHz, 3 kHz, 8 kHz was used. Each stimulus lasted 6 seconds and was delivered via an MR compatible audio system, MR-Confon HP SC01 (MR Confon GmbH, Magdeburg, Germany). Stimuli were presented in pseudo-random order (10 stimulations for each frequency) during single measurements that contained 152 dynamical volumes (with a repetition of 8 s) and lasted 20 minutes (Fig. 4). Volunteers were instructed to press an answer button every time the acoustic stimulus was heard.

Functional MRI data was evaluated in SPM8 (Wellcome Trust Centre for Neuroimaging, London, UK). Preprocessing included realignment, slice timing, smoothing to 6x6x6 mm^3^ and normalization to MNI-152 space. Individual statistic maps were calculated using a general linear model (GLM) for each stimulation frequency. The group statistic (t-test, p = 0.05 FWE correction) was used to compare the responses of each group to all frequency stimulations. The difference between groups was tested with an un-paired two-sample t-test, uncorrected threshold set at p = 0.001. The lateralization index (LI) was calculated by the bootstrap method [20, 21] on different maps (e.g. MP > YC). The default parameters of the SPM LI toolbox were used for LI calculations: sub-sample size k = 25%, minimum sample size 5, maximum sample size 10 000 and only voxels from temporal lobes were used.

## Results

**Fig 5:**
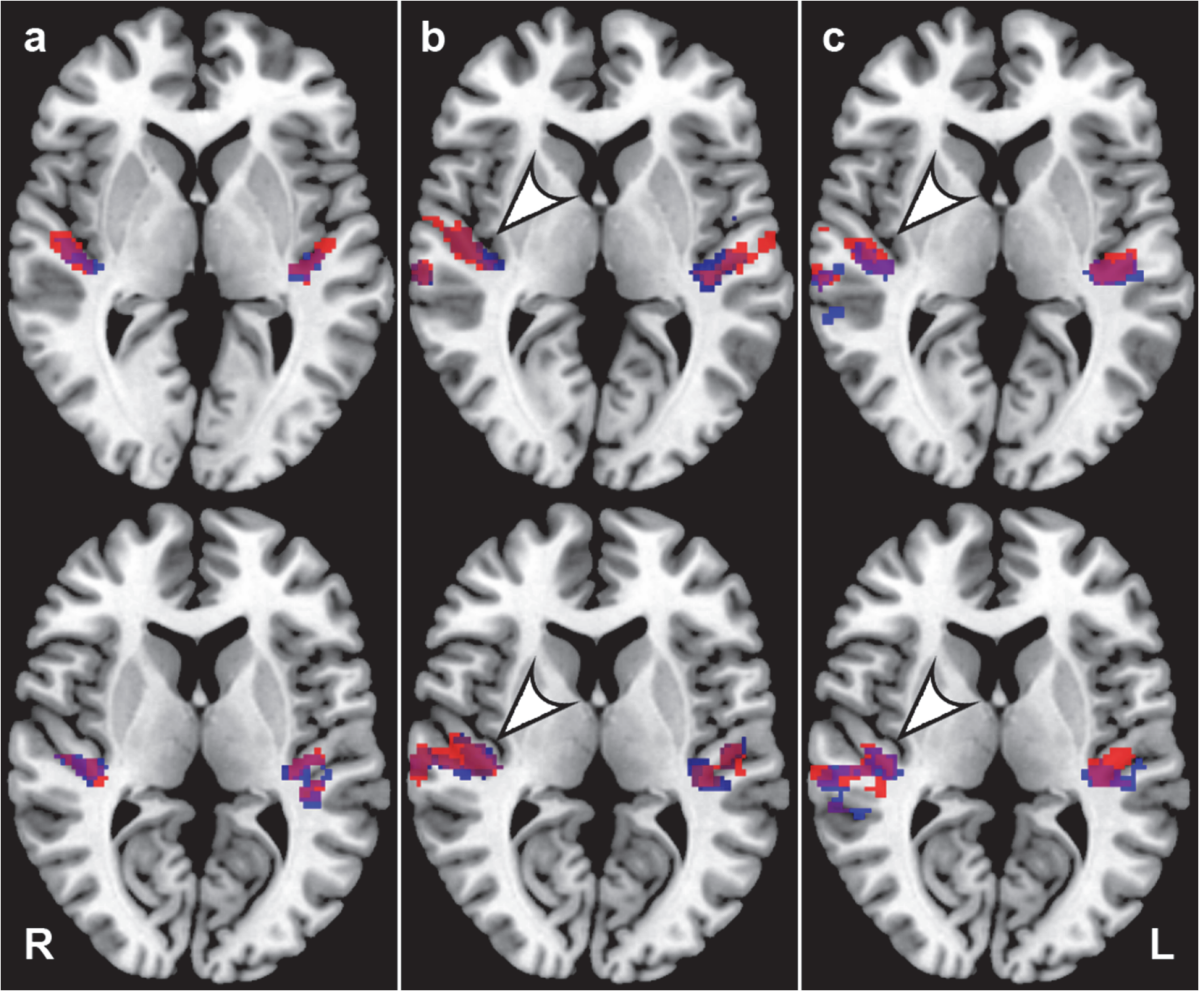
Averaged cortical activity evoked by acoustic stimulation examined by fMRI in all 3 groups. A—YC, b—MP, c—EP; group statistics, p = 0.05 with FWE correction; red—pink noise centered at 350 Hz and 700 Hz; blue—pink noise centered at 1.5 kHz, 3 kHz and 8 kHz. The arrowheads accentuate an increase in the right AC activation in both elderly groups.

Significant activation (p = 0.05, FWE correction) was observed in all subjects at lower and middle frequencies (Fig. 5). In 9 subjects (3 from MP, 6 from the EP group) minimal or no activation during the highest frequency stimulation (8 kHz) was found either on one or both sides (p = 0.05, FWE correction). All subjects confirmed hearing the acoustic stimulation (even the 8 kHz stimulus which did not evoke the cortical response) by pressing the response button.

Although the analysis of the tonotopic organization of the AC was not the aim of this study, the tendency of the activated AC region to shift in a medial direction with increasing frequency of the stimulus was observed (there is no statistic to support this observation)(Fig. 5). The number of activated voxels decreased with increasing stimulation frequency in all groups, but the decrease was more prominent in both elderly groups with the most significant drop in EP at 8 kHz. Low frequency (≤1.5 kHz) stimulation produced a larger amount of active voxels in the elderly than in YC. In contrast, high frequencies (8 kHz and partially also 3 kHz) evoked greater activation in YC than in the elderly (Table 1).

**Table 1:** Number of activated voxels evoked by acoustic stimulation in all three groups in the left and right temporal lobe.

group	frequency	lateral	K	T	Z-score	x	y	z
**YC**	350 Hz	right	315	15.04	6.65	42	-28	12
			left	203	13.16	6.33	-36	-24	6
	700 Hz	right	122	11.33	5.97	40	-26	12
		left	269	14.14	6.51	-38	-22	4
	1.5 kHz	right	200	13.55	6.4	42	-26	12
		left	208	15.92	6.78	-34	-24	6
	3 kHz	right	109	12.32	6.17	44	-18	4
		left	183	13.99	6.48	-38	-22	0
	8 kHz	right	122	12.3	6.17	38	-26	10
		left	135	12.13	6.13	-36	-24	6
**MP**	350 Hz	right	276	12.55	6.22	46	-22	8
			left	227	10.56	5.79	-52	-18	2
	700 Hz	right	503	13	6.3	68	-26	8
		left	179	10.63	5.81	-48	-18	2
	1.5 kHz	right	434	14.4	6.55	68	-24	8
		left	326	14.48	6.56	-46	-16	0
	3 kHz	right	99	10.27	5.72	40	-28	12
		left	96	9.82	5.61	-46	-20	0
	8 kHz	right	107	10.1	5.68	54	-24	14
		left	49*	8.89	5.36	-42	-32	12
**EP**	350 Hz	right	771	13.78	6.44	52	-14	2
			left	207	16.06	6.8	-40	-24	6
	700 Hz	right	199	10.28	5.72	58	-12	-2
		left	228	13.85	6.45	-36	-24	10
	1.5 kHz	right	419	10.8	5.85	40	-24	8
		left	189	13.39	6.37	-36	-26	8
	3 kHz	right	419	13.33	6.36	42	-22	6
		left	215	11.83	6.07	-36	-26	8
	8 kHz	right	0					
		left	0					

Active voxels (at least 40 (* 20) connected voxels) survive the statistical significance p = 0.05 with applied FWE correction for multiple observations; k is the number of activated voxels; T, Z score are statistical indices for SPM; x, y, z are coordinates in MNI-152 space.

**Fig 6:**
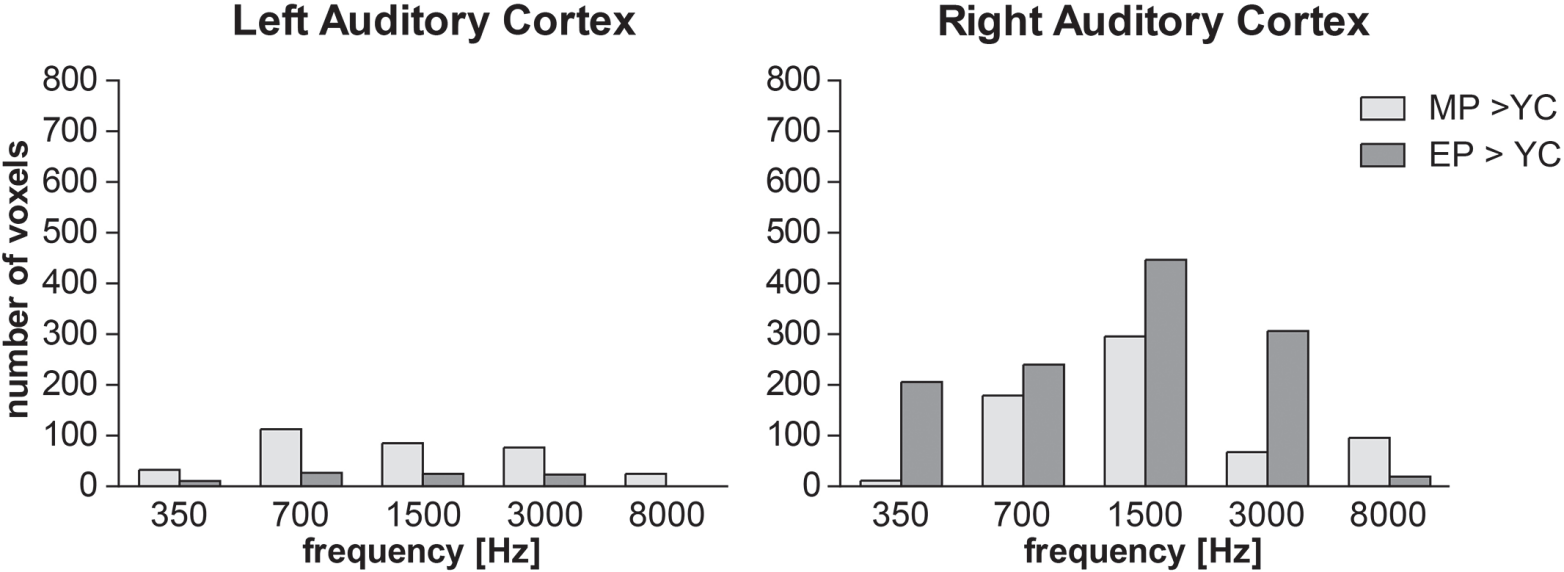
A comparison of temporal lobe activation (difference maps (from temporal lobe) of group statistics) between MP vs. YC and EP vs. YC, shown as the difference in the amount of activated voxels (threshold set at p = 0.001 uncorrected).

To examine the effect of presbycusis, the activated regions of AC were compared between all three groups with the use of group statistics. In the AC, both elderly groups showed significantly higher activation (illustrated by the number of activated voxels with significantly different activation) than YC (Fig. 6). A comparison of MP vs. EP showed no statistically significant difference. For statistical significance uncorrected p = 0.001 was used.

The results of the fMRI investigation indicated an increased activation with ageing that seems more prominent in the right temporal lobe. This is true under all conditions with the exception of 8 kHz stimulation in the EP group. However, the statistical approach used is strongly dependent on a pre-selected statistical threshold used for the creation of group maps. Therefore, the lateralization index (LI) was calculated by means of the bootstrap method published by Wilke et al. [20]. The LI showed only a slightly higher activation of the right temporal lobe at low frequencies (350 and 700 Hz) in the MP group where values were close to 0. Under all other conditions the LI values were negative and confirmed right temporal lobe dominance with ageing. The results of the bootstrap statistics, thus, confirmed right temporal lobe lateralization in both elderly groups (Table 2).

**Table 2:** The lateralization index (LI) was calculated using the bootstrap method by comparing the difference maps of temporal lobe activation (MP vs. YC, EP vs. YC).

Group diff	350 Hz		700 Hz		1.5 kHz		3 kHz		8 kHz	
	mean	W mean	Mean	W mean	mean	W mean	mean	W mean	mean	W mean
**MP>YC: LI**	**0.065**	**0.014**	**-0.15**	**-0.14**	**-0.34**	**-0.47**	**-0.28**	**-0.27**	**-0.34**	**-0.4**
**std**	0.17		0.09		0.22		0.14		0.1	
**lowest**	-0.22		-0.27		-0.79		-0.47		-0.52	
**highest**	0.5		0.17		-0.09		0.19		-0.15	
**EP>YC: LI**	**-0.26**	**-0.44**	**-0.34**	**-0.5**	**-0.41**	**-0.55**	**-0.44**	**-0.58**	**-0.43**	**-0.6**
**std**	0.34		0.29		0.12		0.24		0.29	
**lowest**	-0.92		-0.92		-0.88		-0.81		-0.9	
**highest**	0.04		-0.05		-0.11		-0.16		-0.1	

Comparison of MP and EP showed no difference. LI results are shown as mean and weighted mean values for each stimulation frequency. Negative values demonstrate right laterality.

Analysis of the dependence of evoked cortical activity on auditory threshold, with PTA set as the regressor, showed no significant results (group statistic, F test, FDR correction) in any of the three groups.

## Discussion

The aim of our study was to characterize functional changes in the auditory cortex occurring in the ageing population in comparison with young controls. It is generally assumed that the presbycusis has both peripheral [22] and central [23] components. The age-related peripheral deterioration is well documented in animal models [24] and also in humans [25]. The central components in humans are more difficult to identify because of the overlap of several age-related changes, such as deterioration of cognitive functions [25, 26], decreases in gray and white matter [27] as well asin the levels of certain neurotransmitters [28]; all of these may influence to some extent the functional processing of auditory signals. Another variable affecting the central processing of auditory information is the degree of complexity of the auditory stimuli. In the elderly population, a complex stimulus such as speech activates the non-auditory cortex to a much greater extent than in the young population [29]. Therefore, we chose simple auditory stimuli (pink noise), which would not require further processing (e.g. as in the case of semantic processing of speech) and would reveal the central component of the presbycusis without contributing to any cognitive decline.

### Auditory function

To assess the function of the auditory periphery, pure tone hearing thresholds in the extended frequency range and TEOAE and DPOAE were recorded. Elderly subjects were divided into MP and EP groups based on their hearing thresholds, which gradually increased with increasing frequency and started to exceed the physiologic range of hearing at a level of 4 kHz for MP and a level of 1.5 kHz for EP. In contrast with audiometry regularly conducted in clinical practice, we also examined frequencies above 8 kHz, where the difference between the two elderly groups became even more apparent. The elevation of hearing thresholds was supported by the examination of OAEs that showed more prominent decreases in the presence and amplitudes of TEOAEs and DPOAEs in the EP group than in MP. Although we could not confirm the change in the right ear dominance caused by ageing [30], the trend is present and is most probably due to the low presence of OAE in elderly subjects. The decrease in the right ear dominance might partially explain the observed increase in the right AC activation. Compromised speech perception in background noise is a prominent symptom of presbycusis, that can be only partially explained by an elevation in hearing [31]. Another factor regarding the decrease in speech perception, which points toward a central component of presbycusis [32], is altered inhibition [33, 34] resulting in compromised detection of short temporal changes [15] and cognitive decline [23]. The altered inhibition, commonly described as the “inhibition deficit hypothesis”, also affects other cognitive domains such as reading [35], verbal learning and fluency [36, 37] as well as reasoning [38, 39].

### Auditory cortex in presbycusis

At the level of the AC, several changes accompanying ageing were previously reported. The structural differences between the young and ageing subjects, studied with MRI, illustrated a loss of myelinization and a degradation of neuronal fibers along the auditory pathway [40, 41], changes which were also observed within the white matter under the AC [41]. Similar findings have also been described in other cortical areas accompanying a general cognitive decline in the elderly [42].

In our previous studies [28, 41] the comparison of two elderly groups (with identical criteria as MP and EP) showed no significant differences between them in structural changes of the grey and white matter of the AC, although both differed from the young control group. Similar age-related changes, that of no differences between the EP and MP groups, but significant differences between both elderly groups and YC, were present when the levels of metabolites (glutamate, N-acetylaspartate, lactate) were compared in the AC [28]. The results of both papers suggest that the peripheral component of age-related hearing loss has only a minimal effect on the structural and neurochemical changes in the AC of the elderly population; however the techniques used (diffusion tensor imaging, MR morphometry and MR spectroscopy) did not directly measure the activity in the AC altered by peripheral hypo-function. However, the finding that ageing is accompanied by increased AC activation should not be generalized to other sensory cortical areas, since there are reports that show age-related decreases of cortical activation for visual and sensorimotor tasks [43].

Activation of the AC in both elderly groups, as measured by fMRI, did not show any significant differences between the MP and EP groups in our experiments. A large decrease in the extent of AC activation was observed with increasing frequency in all three groups, which was more pronounced in both elderly groups. This frequency related decrease observed is in agreement with the audiological findings of a decrease in the peripheral function at frequencies above 1.5 kHz. Interestingly, at low frequencies (≤1.5 kHz), where the difference between auditory thresholds was minimal, the activated volume of the AC was significantly larger in EP and MP than in YC. This finding supports the presence of a non-peripheral component of presbycusis at the level of the AC.

The AC shows hemispheric functional differences. In a simplified manner, the left AC is responsible for processing the temporal parameters of sound whilst the right AC detects changes in pitch [44, 45]. Speech sounds predominantly activate the left AC, and music sounds the right [46]. Overall, in language processing the left AC dominates over the right AC independent of left/right-handedness [47]. This is also true in morphometric characteristics of the AC, where the size advantage of the left planum temporale over the right varies between 64 and 82% [48]. As Salvi at al. [49] have shown, the extent of cortical activation, especially in non-auditory areas, depends also on the difficulty of the auditory task.

In more detail, within the human AC are regions more specialized for temporal processing (the central part of the Heschl’s gyrus) that are independent of lateralization; an exception to this is the antero-lateral portion of the planum temporale (belt region), which on the left side specializes in temporal processing whereas on the right side it is responsible for spectral cues [50]. Similar hemispheric specialization is already present in lower mammals such as rats, where a lesion to the right AC, in comparison to the left, causes significant worsening of discrimination of frequency modulated stimuli [51], whilst a lesion to the left AC causes decreased discrimination of temporal parameters [52].

Our results show an increased involvement of the right AC in the process of ageing. The activation of AC increases with age, more prominently in the right AC with the exception of stimulation by 8 kHz, which corresponds with hearing loss at this frequency. Overall, in both elderly groups, the activation of AC is larger in the right than in the left AC, which is opposite to the finding in the YC group. The increased activation of the right AC in processing simple auditory stimuli is clearly an effect of the ageing brain that does not majorly impact the altered periphery. In the context of our previous results from morphometric analysis of the AC [41], the fMRI data confirmed the change in the function of the left and right AC. Although the morphometric changes of the grey matter in the AC are not side specific and therefore do not show an effect of an increased employment of the right AC in the elderly, there is a clear rightward asymmetry in the white matter under the AC in the ageing population [41]. This corresponds with increased activation of the right AC in the ageing population observed in our present experiments. Another explanation for the increased activation of the AC in the elderly groups compared to YC may be decreased inhibition that appears with aging. In animal experiments, a decrease of GAD (precursor of GABA) [53] and Ca binding proteins specific for inhibitory interneurons [54] accompany ageing and directly affect the electrophysiologic features of neuronal responses [55] in the aged population.

### Central presbycusis

The idea of central presbycusis [23, 56–58] is founded on two basic hypotheses, a direct and an indirect hypothesis as well as a combination of both. The direct hypothesis supposes pure central changes that occur without concomitant peripheral lesions [59, 60]. The indirect hypothesis is based on the central effect of the peripheral lesion displayed as an increase in the auditory threshold [61]. Several studies have shown that degraded peripheral stimulation [62, 63] or a long sensory deprivation [23, 64] may cause cognitive hypo-function.

As previously stated, ageing is accompanied by deficits in cognitive functions that in general consist of the deterioration of the speed of processing, attention, perception, working memory, cued and free recall [65]. A decrease in cognition has also been linked to a reduction of volume and activity in the hippocampus [66]. Changes in the activity related to cognitive control with ageing are also present in the prefrontal cortex, especially in its dorsal part [67]. In the processing of speech, several of these components are used for attending the signal, temporally storing the acoustic information, identifying the phonemes, and comparing them with meaning [68]. In general, it can be assumed that a decline in sensory activation can be accompanied by an increase in the recruitment of more general cognitive areas [69]. Wong et al. [70] showed that for word processing in noise, which is the main difficulty of presbycusis, older subjects use a broader network (including the frontal lobe regions) to a greater extent than young controls. The increased employment of frontal lobes in the processing of complex sounds in the elderly is also present during memory processes [71] and response inhibition tasks [72]; it is probably a means of compensating for the lost ability to inhibit irrelevant stimuli such as background noise. Eckert et al. [29] also showed an increase in the activity of the middle frontal gyrus during speech stimulation that correlates with a structural decline in the temporal lobe of the elderly.

Our data together with the above described cognitive changes suggest that the presbycusis consists of several components which include a purely peripheral component (decreasing the extent of activated AC during high frequency stimulation), as well as changes at the level of the AC (increased right AC involvement, greater activation of the AC in general) and increased involvement of non-auditory (prefrontal) cortical areas in the elderly.

## Conclusions

In general, our audiological data indicates the existence of different degrees of presbycusis in the ageing population. The difference in the function of the outer hair cells remains the main peripheral factor characterizing the differences between two categories of presbycusis, MP and EP, resulting in the increased thresholds of EP. At the level of the AC and based on the status of their auditory periphery, the differences seen in the elderly groups were minimal, although the activation of the AC by frequencies with increased thresholds clearly diminished. Therefore, we can argue that the effect of presbycusis at the level of the AC is a combination of ageing and altered periphery.

The main effect demonstrated in our study was the increased activation of the right AC and larger activation of the AC overall in both elderly groups in comparison with young controls. This finding indirectly supports the theory of cognitive decline as the basis of central presbycusis, which requires an increased recruitment of more general cognitive areas for the processing of complex tasks.
